# Diabetes remission of bariatric surgery and nonsurgical treatments in type 2 diabetes patients who failure to meet the criteria for surgery: a systematic review and meta-analysis

**DOI:** 10.1186/s12902-023-01283-9

**Published:** 2023-02-22

**Authors:** Xiaoying Zhou, Chunping Zeng

**Affiliations:** Department of Endocrinology and Metabolism, The Fifth Affiliated Hospital of Guangzhou Medical University No.621, Gangwan Road, Guangzhou, 540700 People’s Republic of China

**Keywords:** Diabetes remission, Bariatric surgery, BMI<35kg/m²

## Abstract

**Background:**

The efficacy of bariatric surgery in moderate and severe obesity patients to reach diabetes remission is clear, but for mild obesity patients, the choice of surgical and non-surgical treatment is still unclear. This study we aim to compare the effect of surgical and nonsurgical treatment on patients BMI < 35 kg/m^2^ to reach diabetes remission.

**Method:**

We searched relevant articles publish between Jan 1,2010 and Jan 1, 2023 in the following databases: Embase, PubMed/MEDLINE, Scopus, and Cochrane Library. We got the OR, MD and P-value using random effect model to compare the efficiency between bariatric surgery and nonsurgical treatment on diabetes remission, the reduction of BMI, Hb1Ac and FPG.

**Results:**

In 7 included studies including 544 participants, bariatric surgery is more effective than non-surgical treatment to reach diabetes remission [OR 25.06, 95%CL 9.58–65.54]. Bariatric surgery more likely resulted in significant reductions in HbA1c [MD -1.44, 95%CL (-1.84)-(-1.04)] and FPG [MD -2.61, 95%CL (-3.20)-(-2.20)]. Bariatric surgery may resulted in reductions in BMI [MD -3.14, 95%CL (-4.41)-(-1.88)], which more significant in Asian.

**Conclusion:**

In type 2 diabetes patients who BMI < 35 kg/m^2^, bariatric surgery is more likely to achieve diabetes remission and better blood glucose control than nonsurgical treatment.

## Introduction

Type 2 diabetes mellitus (T2DM) has been regarded as a chronic disease characterized by hyperglycemia caused by the interaction of genetic factors and environmental factors. According to data released by the International Diabetes Federation (IDF), the global prevalence of diabetes among people aged 20–79 is estimated at 10.5% (536.6 million people) in 2021, and is expected to rise to 12.2% (783.2 million) by 2045 [1]. T2DM has high incidence rate, morbidity and mortality, causing a heavy burden on individual families and society.

The 2021 ADA consensus statement updated the recommended definition of diabetes remission: HbA1c < 6.5% for at least 3 months after suspending hypoglycemic drug [2]. If there are factors that affect the accuracy of HbA1c detection, the surrogate indicator can be use: the average HbA1 < 6.5% estimated by continuous blood glucose monitor or fasting blood glucose < 7.0 mmol/L [3]. Diabetes requires long-term treatment with antidiabetic medications, which is essential to prevent acute complications and reduce the risk of long-term complications. However in recent years, this statement is gradually changing with the continuous accumulation of evidence-based medicine [4]. When patients reach diabetes remission, they can free from hypoglycemic medications for some time and delay disease progression.

It is known that three ways to reach diabetes remission. There is clear evidence that intensive lifestyle management and bariatric surgery can promote the remission of T2DM by significantly reducing body weight [2, 5, 6]. In previous primary care-led weight management study, 46% of patients with T2DM reached diabetes remission [2]. For bariatric surgery, 72% of T2DM patients can achieve diabetes remission after 2 years [7]. In addition, latest clinical evidence supports that short‑term intensive insulin therapy (SIIT) also be used to reach T2DM remission [8]. For 2 weeks of SIIT treatment, 42% of patients with T2DM reached diabetes remission after 2 years [9].

According to NIH (National Institutes of Health) standard, type 2 diabetes patients who BMI above 35 kg/m2 are eligible for bariatric surgery. However this standard was formulated 20 years ago and have not updated, and now the mortality and complications of surgery have been reduced compared with 20 years ago [10, 11]. Indication for bariatric surgery in patients with BMI < 35 kg/m^2^ is controversial, and no guidelines currently recommend bariatric surgery for non-obese patients. Therefore, it is necessary to study diabetes patients with BMI < 35 kg/m^2^. Current nonsurgical treatments for patients with BMI < 35 kg/m^2^ are often ineffective in achieving significant long-term weight loss and diabetes remission. There is new evidence to support that the mild obese diabetic patients who suffered bariatric surgery are more able to reach diabetes remission than those who had non-surgical therapy [12]. This article aims to evaluate the diabetes remission, weight loss and blood glucose control of patients with BMI < 35 kg/m^2^ who had bariatric surgery and other nonsurgical treatments, and provide clues whether to have bariatric surgery for patients who BMI do not meet the surgical criteria.

## Methods

### Search strategy and selection criteria

This systematic review and meta-analysis follow guidelines established by the Preferred Reporting Items for Systematic Reviews and Meta-Analyses (PRISMA) statement and was registered at International Prospective Register Systematic Reviews (number CRD42022306363).

We searched relevant literature publish between Jan 1, 2010 and Jan 1, 2023 in the following database: Embase, PubMed/MEDLINE, Scopus, and Cochrane Library. Articles were identified by combination of keywords: "bariatric surgery" OR "metabolic surgery" OR "diabetic surgery" AND "BMI < 35 kg/m^2^" OR "mild obese" AND “diabetes remission” OR “remission of diabetes”. The complete search was: (bariatric surgery [Mesh Terms] OR metabolic surgery [Text Word] OR diabetic surgery [Text Word]) AND (BMI < 35 kg/m^2^ [Mesh Terms] OR mild obese [Text Word]) AND (diabetes remission [Mesh Terms] OR remission of diabetes [Text Word]).

We included all potentially eligible studies for review written in the English language. Two independent investigators (ZX, LT) reviewed the literature. We screened the articles through database search, screening title and abstract then inclusion remaining studies for detailed assessment. Research with design research defects, statistical method errors that cannot be corrected and unable to obtain appropriate outcome indicators will be excluded. And the included articles were read in full and assessed by two investigators. Finally, disagreements will be resolved by a third investigator (ZC). The included literature is explained by Cochrane bias risk assessment.

We extracted the following data from included studies: year of publication, sample size, country, type of study, type of bariatric surgery, type of nonsurgical treatment, baseline mean BMI (kg/m^2^), number of participants reach diabetes remission, change in BMI (mean, SD), change in glycosylated hemoglobin(HbA1c) (mean, SD), change in fasting plasma glucose(FPG) (mean, SD). In addition, we assessed risk of bias based on PRISMA recommendations.

### Statistical analysis

We use review management V.5.4 for data processing and analysis. We assessed the efficiency between of the bariatric surgery and nonsurgical treatments through four outcomes: number of participants reach diabetes remission, change in BMI, change in HbA1c and change in FPG. We analyzed number of participants reach diabetes remission as a dichotomous variable, using Mantel–Haenszel method to report the difference before and after the intervention. We analyzed change in BMI, HbA1c and FPG as a continuous variable, using Inverse Variance method to reported the difference between the arithmetic mean before and after the intervention. In addition, in the group of change in BMI, we conducted subgroup analysis grouped by ethnicity.

We use Cochrane Q test to assess the heterogeneity of studies, and we can consider that there is no heterogeneity when the *p*-value < 0.1. We also use I^2^ test to assess the heterogeneity of studies, and heterogeneity is acceptable as long as I^2^ < 50. Sensitivity analysis was carried out by removing individual studies one by one to observe the changes of heterogeneity and deleting the small sample studies that make the funnel diagram asymmetric. Revman5.4 is used to evaluate risk bias and visualize the results into risk of bias graph and risk of bias summary. And we identified possibility of publication bias by funnel plot.

## Results

### Study characteristics

Based on the keyword combination search, we identified 351 articles, and after screening the titles and abstracts and reading the full articles, 7 articles were included for our study (Table 1). The included studies were all published after 2010. The population included Caucasian and Asian with baseline mean BMI between 29-35 kg/m^2^. For nonsurgical treatment, Keong C’s used medical management, Wenhuan F’s combined weight management and antidiabetic drugs and Mohit B’s only used glucagon-like peptide-1 analogues (GLP-1), and all of them used Roux-en-Y gastric bypass surgery (RYGB) as surgical treatment [13–15]. Daniel H’s clinical trial is a randomized controlled study used three different types of surgery and medical weight management as non-surgical treatment [12]. Chih-Cheng H’s clinical trial is a retrospective cohort study used laparoscopic sleeve gastrectomy (LSG) and gastric bypass (GB) as surgical treatment, and medical management as non-surgical treatment [16]. John M’s clinical trial is a randomized controlled study used laparoscopic adjustable gastric banding (LAGB), and multidisciplinary diabetes care as non-surgical treatment [17]. Bruno G’s Retrospective cohort study used duodenal-jejunal bypass (DJB), and standard medical care as non-surgical treatment [18].

**Table 1:** Characteristics of included studies

Study	Year of publication	Sample size	Country	Design	Type of surgery	Medical therapy	Follow-up(Months)	Baseline mean BMI(kg/m^2^)	Definition of diabtes Remission
Daniel H et al	2020	57	America	Randomized Controlled Trial	RYGB(28%), LSG(62%) or LAGB(10%)	Medical weight management	36	32.8 (bariatric surgery) 32.0 (medical therapy)	Not reported
Keong C et al	2017	41	America	Randomized Controlled Trial	RYGB	Intensive medical management	41	32.9	Blood HbA1c < 6.5% (48 mmol/mol) or < 6% (42 mmol/mol) without any antihyperglycemic medication for at least 1-year duration, respectively
Wenhuan F et al	2018	40	China	Retrospective cohort study	RYGB	Weight management and antidiabetic agents	12	33.3 (bariatric surgery) 32.1 (medical therapy)	T2DM required HbA1c less than 6.0% and fasting blood glucose (FBG) less than 5.6 mmol/L, with no use of antidiabetic agents for 1 year
Chih-Cheng H et al	2015	351	China	Retrospective cohort study	LSG(36.5%) or GB(63.5%)	Medical treatment	60	31.0 (bariatric surgery) 29.1 (medical therapy)	HbA1c < 6.0% and 6.0%-6.5% of total hemoglobin [Hb; to convert to proportion of total Hb, multiply by 0.01], respectively, for those who were exempted from any antidiabetic drugs for 5 years
Mohit B et al	2017	70	India	Prospective study	RYGB	GLP-1	12	34.17 (bariatric surgery) 32.67 (GLP-1) 31.33(SGLT2)	Not reported
John M et al	2014	51	Australlia	Randomized Controlled Trial	LAGB	Multidisciplinary Diabetes Care	24	29.0 (bariatric surgery) 29.0 (medical therapy)	serum glucose values were ≤ 125 mg/dL and HbA1c ≤ 6.0%, on free diet and with no antidiabetic medical therapy, controlled when, under the same conditions, HbA1c was ≤ 7.0%, and improved when preoperative HbA1c was steadily reduced by at least 1% with less antidiabetic therapy
Bruno G et al	2012	36	America	Retrospective cohort study	DJB	standard medical care	12	26.1 (bariatric surgery) 26.3 (medical therapy)	Not reported

*RYGB* Roux-en-Y gastric bypass, *LSG* Laparoscopic sleeve gastrectomy, *LAGB* Laparoscopic adjustable gastric banding, *DJB* Duodenal-jejunal bypass, *GLP-1* Glucagon-like peptide-1 analogues

### Meta-analysis results

#### Diabetes remission

**Fig. 1:**
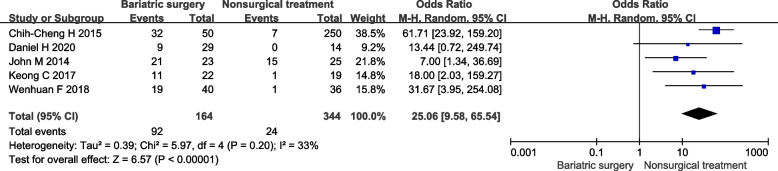
Forest plot of patients reach diabetes remission after bariatric surgery compared to non-surgical treatment

In 7 included studies 5 studies reported number of patients reach diabetes remission. 64.0% (32/50) of participants achieved diabetes remission after surgical intervention and 2.8% (7/250) achieved diabetes remission after non-surgical treatment, which bariatric surgery is more likely to achieve diabetes remission than nonsurgical treatment. Under the random effect model, the OR is 25.06, 95% confidence interval is 9.58–65.54, *P* < 0.00001 (Fig. 1).

#### BMI

**Fig. 2:**
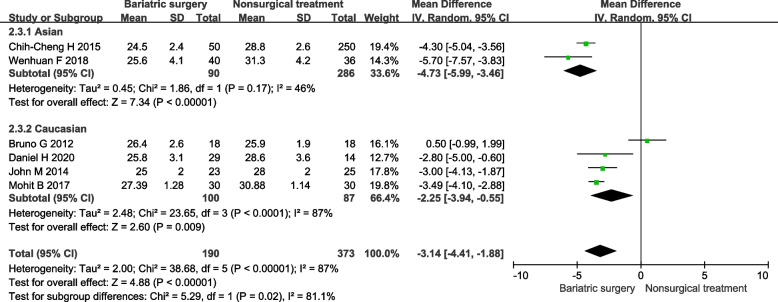
Forest plot of BMI level after bariatric surgery compared to non-surgical treatment

In 7 included studies 6 studies reported the mean BMI before and after intervention. Patients who had bariatric surgery is more significantly lower BMI than non-surgical treatment. Under the random effect model, the MD is -3.81, 95% confidence interval is (-4.55)-(-3.06), *P* < 0.00001 (Fig. 2). After subgroup analysis grouped by ethnicity, the effect was greater for Asians [MD -4.73, 95%CL (-5.99)-(-3.46)] than Caucasians [MD -2.55, 95%CL (-3.94)-(-0.55)].

#### HbA1c

**Fig. 3:**
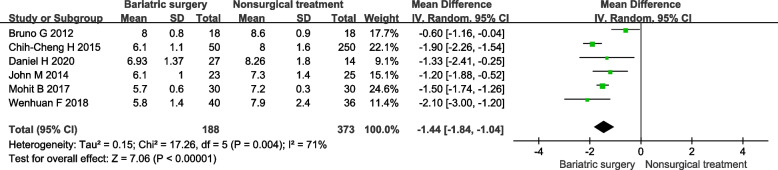
Forest plot of HbA1c level after bariatric surgery compared to non-surgical treatment

In 7 included studies 6 studies reported HbA1c. It shows that bariatric surgery is more significant lower HbA1c than non-surgical treatment. Under the random effect model, the MD is -1.44, 95% confidence interval is (-1.84)-(-1.04), *P* < 0.00001 (Fig. 3).

#### FPG

**Fig. 4:**
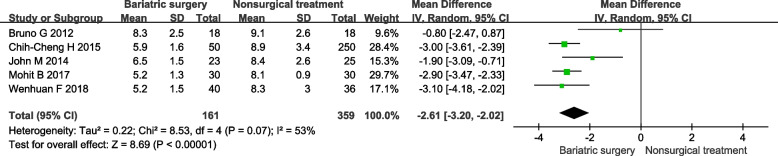
Forest plot of FPG level after bariatric surgery compared to non-surgical treatment

In 6 included studies 4 studies reported FPG. It shows that bariatric surgery is more significant lower FPG than non-surgical treatment. Under the random effect model, the MD is –2.61, 95% confidence interval is (-3.20)-(-2.02), *P* < 0.00001 (Fig. 4).

### Risk of bias in the included study

**Fig. 5:**
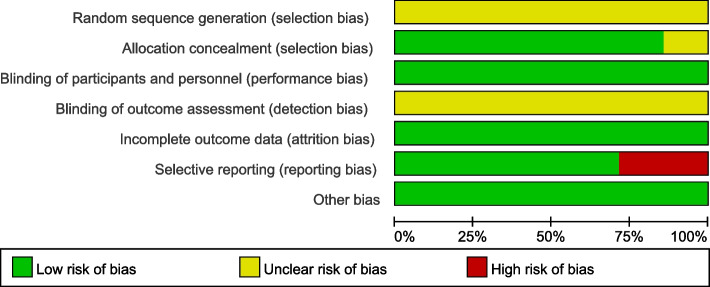
Risk of bias graph of the authors' judgment of each bias risk item included in the study

**Fig. 6:**
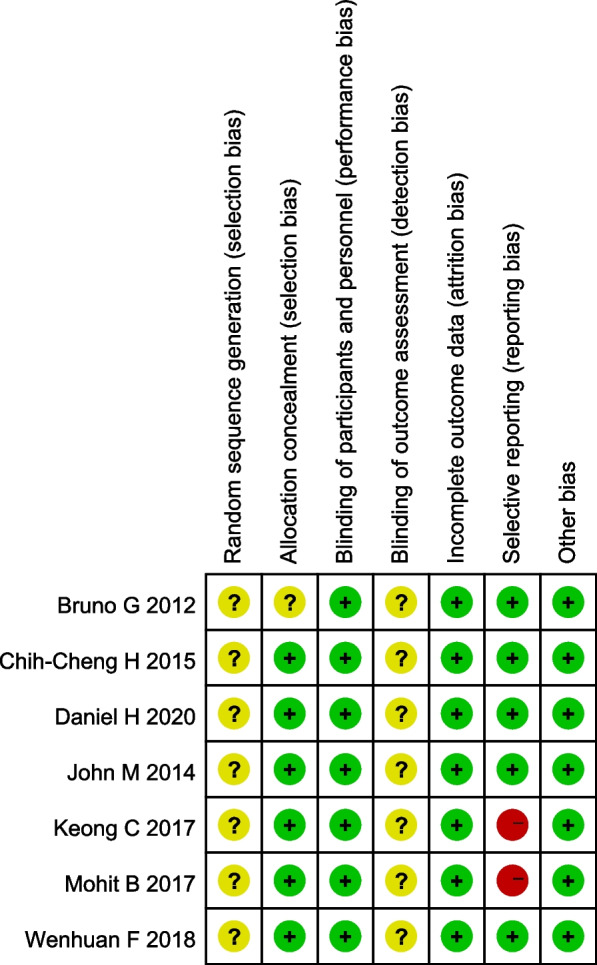
Risk of bias summary of the authors' judgment of each bias risk item included in the study

The risk of bias was particularly high in the domain of selection, detection and reporting bias due to the included studies failure to generate a random sequence, assess the blinding of outcome and report the outcome fully (Figs. 5 and 6).

### Sensitivity analysis and publication bias

Sensitivity analysis was performed to assess the reliability of the results by sequentially repeating the meta-analysis. Our results may be affected by publication bias, for statistically meaningful outcome are published more frequently than meaningless outcome.

## Discussion

Our results demonstrated that bariatric surgery is more efficient than non-surgical treatment in achieving diabetes remission for patients with BMI < 35 kg/m^2^ who failure to reach the criteria for surgery. Although the non-surgical treatment included strict weight management, oral drug therapy and injection of insulin and GLP1, bariatric surgery is superior to reach diabetes remission and more significant reduction in BMI, HbA1c and FPG. The studies have shown that surgical treatment can achieve diabetes remission in obese patients. For G. Ribaric’s meta analysis, the overall T2DM remission rate for surgery group was 63.5% and conventional group was 15.6% [19]. For our meta analysis, the overall T2DM remission rate for surgery group was 56.1% and conventional group was 7.0%. Our T2DM remission rate were slightly lower than G. Ribaric’s studies, and this difference can be explained by low BMI in our patients. For weight loss is central to improving high blood glucose and magnitude of weight improvement was the point of remission in T2DM. Moreover, although the BMI of most patients can be reduced after the completion of bariatric surgery, some patients still have not achieved diabetes remission. This may be related to the basic situation of the patient. The younger the age, the larger the baseline BMI, the higher C-peptide, and the shorter the duration of diabetes, the more likely to achieve diabetes remission [20]. Under the control of these basic situation, the effect of bariatric surgery is still better than nonsurgical treatment. As a consequence, bariatric surgery could be regarded as an effective treatment option for patients with BMI < 35 kg/m^2^.

However we needs to continuously monitor the complications of surgery and blood glucose for a long time, for some patients may relapse hyperglycemia and their baseline BMI is not very high [21]. Overweight or obese patients with T2DM are often accompanied by fatty liver disease. Fatty liver disease causes lipid spillage enter the pancreas from the liver, leading to pancreatic lipid deposition (lipopancreas), thus affecting the β-cell functio. Reducing lipid deposition in liver, skeletal muscle, pancreas and other important organs is important for T2DM remission. Bariatric surgery achieves hypoglycemic effects mainly through weight loss, and is also associated with post-metabolic surgery metabolism and absorption, changes in hunger and satiety, food preferences, and possible energy expenditure [22, 23]. It is known that regardless of treatment, there is a strong correlation between the amount of weight loss and the likelihood of remission of diabetes. Although the patients with BMI < 35 kg/m^2^ who suffer bariatric surgery have limited weight loss, and even fewer in patients with BMI < 30 kg/m^2^, the positive effect on diabetes remission after the bariatric surgery is predictable [24, 25]. Therefore, for patients who BMI < 35 kg/m^2^ or hope to reduce the drug burden, it is feasible to treat with bariatric surgery to lose weight to achieve diabetes remission [26]. However, in studies of patients with BMI < 35 kg/m^2^, the comparison between bariatric surgery and nonsurgical treatment is lacking. The lack of evidence leads to the delay of bariatric surgery for patients with BMI < 35 kg/m^2^. Among the current interventions not strictly limited for BMI, the DiRECT intervention (weight management based on primary care) in diabetes remission is significant [2], and SIIT (short-term intensive insulin therapy) also be recommended to be used in diabetes remission. These two interventions are long-term, strict and standardized, and even need lifelong management. And they have far fewer complications than bariatric surgery. On the contrary, the advantage of bariatric surgery is that it takes effect quickly and does not need long-term strict compliance. These interventions all play a significant role in diabetes remission and each one has its advantage, but it still unknown which one works better in patients with BMI < 35 kg/m^2^.

In the analysis of BMI, there was heterogeneity in the results. We tried to use the method of subgroup analysis to determine which factors are significant sources of heterogeneity. We set up different subgroups including different baseline, diabetes duration and race for subgroup analysis, but the source of heterogeneity still could not be found. And we used the method of trim-and-fill, finding out the source of heterogeneity. When we removed the Wenhuan F’s study, heterogeneity I^2^ is 41%. We compared all the study that we included, finding the decrease of BMI in bariatric group in this study was lower than other studies. It may due to the higher baseline mean BMI or shorter follow up month, which leads to lower decrease of BMI. That may be the reason for the heterogeneity of BMI [14]. Bruno G’s study also the source of heterogeneity in outcome of BMI. It can be explained that the baseline BMI is far lower in this study, and his baseline BMI is 26.3 kg/m^2^ (control group) and 26.1 kg/m^2^ (surgical group). Therefore, the weight loss effect is not obvious in both control group and surgical group, resulting in the heterogeneity. Bruno G’s study supposed that the effect of glucose metabolism is a direct consequence of metabolism rather than a secondary effect of weight loss. However, more possibilities need to be supported by more research [18].

In the analysis of HbA1c and FPG heterogeneity also exists in the results and heterogeneity comes from Bruno G’s study. when Bruno’s study is removed, heterogeneity I^2^ is 34% (outcome of HbA1c) and 0% (outcome of FPG). Both HbA1c and FPG reflect blood glucose control, indicating that compared with other studies, the blood glucose control in this study was slightly poor. However, after 12 months of follow-up, patients still had significantly decrease of blood glucose and HbA1c than before, and after the surgery glucose homeostasis was improved and diabetes resistance was reduced.

Although bariatric surgery has excellent effects on weight loss and blood glucose relief, the complications of bariatric surgery could not be ignored. Existing studies have shown that the incidence of postoperative complications in patients with BMI < 35 kg/m^2^ is 6–20% [27], and in patients with BMI > 35 kg/m^2^ the incidence of major complications is 2–6%, and minor complications is up to 15%. This suggests that the incidence of complications is similar, and the following complications may occur after surgery. The incidence of gastroesophageal reflux after Roux-en-Y gastric bypass is higher than that of other types of surgery [28, 29]. Anemia caused by postoperative malnutrition and trace element malabsorption may also occur after treatment of bariatric surgery [30, 31]. Postprandial hypoglycemia can also occur, especially in Roux-en-Y gastric bypass surgery [32].

We must consider some limitations. First, because the research is published in different years, different studies may have difference criteria in T2DM remission and the follow-up time of each study is also inconsistent. Secondly, so far, bariatric surgery for patients with BMI < 35 kg/m^2^ is still relatively rare, only a small number of clinical trials included in our study. In addition, because the clinical trials we included has incomplete outcome events, our results may be affected by reported bias.

Our finding has revealed that in type 2 diabetes patients with BMI < 35 kg/m^2^, bariatric surgery can achieve better diabetes remission and blood glucose control than nonsurgical treatment. For those baseline BMI > 30 kg/m^2^, the effect of weight loss after bariatric surgery is obvious, but for those BMI < 30 kg/m^2^, the effect of weight loss is not obvious, but good glycemic control can still be achieved. It may relevant to bariatric surgery directly affects glucose metabolism, but the specific mechanism still needs to be explored.

## Data Availability

The datasets analysed during the current study are available. Please contact Dr. Zeng and email is zcp193@163com if the data needed from this study.
